# The impact of feedback literacy on reflective learning types in Chinese high school students: based on latent profile analysis

**DOI:** 10.3389/fpsyg.2025.1516253

**Published:** 2025-05-14

**Authors:** Yuhong Gong, Shang Zhang, Ting Zhang, Xinfa Yi

**Affiliations:** ^1^Faculty of Education, Shaanxi Normal University, Xi'an, China; ^2^Department of Basic Teaching, The Engineering & Technical College of Chengdu University of Technology, Le'shan, China; ^3^Key Laboratory of Modern Teaching Technology (Ministry of Education), Shaanxi Normal University, Xi'an, China

**Keywords:** high school students, reflective learning, latent types of reflective learning, feedback literacy, latent profile analysis

## Abstract

**Objective:**

Existing research has not yet thoroughly explored the mechanism through which feedback influences reflective learning, failing to effectively guide teaching practice. This study shifts the research focus from a singular feedback to a comprehensive exploration of feedback literacy, and delves into the internal dynamics of groups to investigate the sub-group characteristics of reflective learning. It aims to provide more detailed empirical evidence, as well as more targeted and operational improvement recommendations for reflective learning practice.

**Methods:**

this study has developed a research design that encompasses latent variable profile analysis and subsequent analyses. A total of 704 Chinese high school students (45.31% male, 54.69% female) effectively participated in the surveys using the Feedback Literacy Scale and the Reflective Thinking Level Questionnaire, along with standardized academic examinations organized by their schools.

**Results:**

the LPA results indicated the presence of four latent types of reflective learning among Chinese high school students, named as follows: the Low Reflective Learning Group (LRLG, 22.9%), the Low Habits-high Understanding Learning Group (LHHULG, 15.6%), the Moderate Reflective Learning Group (MRLG, 46.3%), and the High Reflective Learning Group (HRLG, 15.2%). It revealed that feedback literacy, age, grade, and gender are closely correlated with the latent types of reflective learning. Meanwhile, a close correlation between the latent types of reflective learning and achievements in Mathematics and English.

**Conclusions:**

students in different latent types of reflective learning groups exhibit distinct characteristics in habitual behavior, understanding, reflection, and critical reflection. As age and grade level increase, the number of students in the HRLG decreases; Males tend to HRLG, MRLG and LHHULG, while females tend toward LRLG. The elicitation, enactment, commitment, and readiness within feedback literacy can significantly influence the latent type grouping of reflective learning, and compared to the LRLG, the MRLG, LHHULG, and HRLG exhibit better performance in English and mathematics. The conclusions of this study not only help educators understand the characteristics of subgroups within latent types of reflective learning but also guide teachers in utilizing the identified relationship between feedback literacy and latent types of reflective learning to implement differentiated instruction or personalized guidance.

## 1 Introduction

Feedback can trigger students' reflective thinking and reflective learning, which in turn affects academic performance and promotes the development of higher-order competencies (Zhang et al., 2024; Loka et al., 2019). However, in actual teaching practice, feedback has not produced the desired effects on students' reflective learning. Some research has shown that students' reflective learning abilities still need further development. Peltier et al. (2005) noted that not all students can spontaneously and effectively carry out reflective learning, as issues like insufficient reflective awareness and surface-level reflection exist. Yaacob et al. (2020) found that most students' reflective abilities have not reached a critical level and they lack self-regulation abilities. There are many reasons for this, not least of which is that existing research on feedback and reflective learning is not deep enough. In previous research, feedback was mostly seen as a teaching tool, like teacher-student or peer feedback (Sortkær and Reimer, 2022), or a teaching strategy, such as positive or negative feedback (Garrote et al., 2024), focusing on its correlational relationship with learning outcomes, rather than conceptualizing it as a developmental competency to investigate its impact on fostering reflective learning processes. Feedback literacy, developed in student feedback practices, is crucial for students to understand and absorb evaluative discourse and suggestive discourse, and to improve their subsequent learning (Zhang et al., 2024). Thus, conducting empirical research on the relationship between student feedback literacy and reflective learning is of great significance for guiding teaching practice. The introduction of the concept of student feedback literacy (Carless and Boud, 2018) and the development of measurement tools (Zhan, 2022) have made it easier for us to carry out this study. Moreover, in previous studies, the researchers have focused on the overall reflective learning characteristics of the subject group (Mauri and Neiva de Figueiredo, 2025), and rarely explored the sub-group characteristics within the subject group (Wong et al., 1995; Radović et al., 2023). Wong et al. (1995) categorized reflective learning into subtypes such as non-reflection, reflection, and critical reflection based on the depth of cognitive engagement, and Radović et al. (2023) categorized it into reflection-prompted vs. comprehension-prompted groups according to external trigger intensity. While variable-centered classification research remains necessary, relying on theoretical categorical grouping often inadequately reflects the subgroup characteristics of reflective learning in authentic contexts. Therefore, person-centered classification research on reflective learning subgroups requires further exploration.

Latent profile analysis (LPA) is a person-centered statistical technique that identifies unobserved subgroups within populations by analyzing patterns across variables. It also facilitates model-based assumption testing and validation, enabling researchers to explore differences in predictor variables across latent classes and examine how these classes perform on outcome variables. Given the persistent limitations of existing research in effectively addressing practical instructional challenges and the compelling need for more in-depth investigations, this study aims to: apply LPA to identify latent profiles of reflective learning among Chinese high school students, examine the demographic and feedback literacy predictors of these latent profiles, and investigate the impact of reflective learning latent types on academic performance in Chinese, Mathematics, and English. This study offers teachers strong support to deeply understand reflective learning latent types among high school students. It also clarifies how to rely on feedback literacy to develop precise teaching strategies and interventions for improving students' reflective learning abilities.

## 2 Literature review

### 2.1 Student feedback literacy

Student feedback literacy involves the knowledge, skills, and attitudes required to interpret and apply feedback to improve learning (Carless and Boud, 2018). Feedback comprises evaluative discourse highlighting gaps between current performance and expected goals, as well as suggests discourse proposing strategies to bridge these gaps (Hattie and Clarke, 2018; Zhang et al., 2024). Evaluative discourse disrupts cognitive equilibrium by exposing discrepancies between students' self-perceptions and external expectations, potentially triggering cognitive dissonance, interpersonal tensions, or emotional conflicts. Students need understanding, reflection, and critical thinking to turn these conflicts into problem situations, spurring inquiry-based learning activities (Dewey, 1933; Zhang et al., 2024). Strong feedback literacy can trigger self-critical reflection, resolve cognitive or interpersonal conflicts, and thereby initiate deeper learning processes. At the same time, suggestive discourse provides problem-solving strategies and supportive guidance, but students require the capacity to decode and assimilate such feedback information to improve their learning (Carless and Boud, 2018; Zhang et al., 2024). Strong feedback literacy enables individuals to actively embrace suggestive discourse, recognize the significance of strategies and guidance in feedback, and even critically alter deeply entrenched beliefs, thereby guiding oneself toward deeper learning. In summary, feedback literacy, as a capability to absorb and comprehend comments and suggestions, can provoke students' cognitive reappraisal (reflection) and critical thinking, thereby facilitating deeper learning (Zhang et al., 2024).

Simultaneously, Zhan (2022) built a six-level feedback literacy framework based on prior feedback capacity and handling research, and developed a tool for assessing student feedback literacy. The model includes the following levels: feedback elicitation involves actively seeking support from teachers, peers, and artificial intelligence to benchmark feedback actions, identify issues, and glean problem-solving strategies. Feedback processing refers to analyzing and judging critical remarks, extracting key information, and thus clarifying positions and improving actions. Feedback enacting means carrying out goal adjustment, plan formulation, monitoring implementation and strategy management based on feedback information. Feedback appreciation involves thinking from others' perspectives, discerning the intentions behind feedback, assessing strengths and weaknesses, and absorbing strategies for improvement. Feedback readiness refers to maintaining an open-minded and courageous attitude, acknowledging mistakes and accepting suggestions. Feedback commitment involves taking decisive improvement actions, adjusting to learn strategies in a timely manner, overcoming challenges, and daring to innovate. Using this conceptual framework, this study will analyze the effects of feedback literacy dimensions on reflective learning subgroups and identify effective strategies for enhancing students' reflective learning abilities. Applying this conceptual framework, this study will conduct in-depth analyses of the impacts of feedback literacy dimensions on reflective learning subgroups and identify effective strategies to promote the development of students' reflective learning competencies.

### 2.2 Reflective learning

Reflective learning is an uncertainty-driven, internally initiated reflective practice, through which learners engage in critical examination and problem-solving of their learning experiences. This process facilitates the personalized construction of knowledge and deepens their understanding of meaning, ultimately leading to a profound reconstruction of existing conceptual frameworks and perspectives (Boyd and Fales, 1983). It operates through four hierarchical dimensions: habitual behavior (automatic reliance on prior knowledge), understanding (logical integration of information), reflection (linking theory to practice), and critical reflection (challenging assumptions to redefine problems; Kember et al., 2000; Radović et al., 2023). In Dewey (1933)'s view, reflective learning originates from uncertain situations, proceeds through inquiry, it finally culminates in grounded assertions (determined situations). In the design of uncertain teaching situations, feedback can provide comments, reveal gaps, and is highly likely to provoke conflict and deep reflection, thereby forming problems. During the inquiry process, feedback can offer more suggestions such as strategies and support, which are highly conducive to guide the formation of hypotheses for solving problems. In the formation of definitive conclusions, feedback can provide support to adjust the gap between ideals and reality, guide students to carry out the testing process to the end and form grounded assertions.

Notably, this interplay between feedback and reflection manifests differently across learners, contributing to heterogeneous reflective patterns (Peltier et al., 2005; Farahian et al., 2021; Coppens et al., 2023). Students' reflective learning exhibits individual differences and is characterized by latent subgroup features. Reflective learning can be categorized into subtypes such as non-reflection, reflection, and critical reflection based on the depth of cognitive engagement (Wong et al., 1995), and classified as reflection-prompted vs. comprehension-prompted groups depending on the intensity of external triggers (Radović et al., 2023). However, classification approaches grounded in singular dimensional or generalized learner characteristics, while partially capturing individual features of reflective learning, are limited in accounting for the complex interactions between dimensions inherent to students' reflective process. Latent Profile Analysis (LPA) is a mixture model that assumes a population consists of several subgroups or latent classes, each showing unique response patterns to observed variables (Yalçin et al., 2021). Based on this, we propose the Hypothesis 1: high school students' reflective learning can be characterized by multiple latent profile types, each with distinct features.

Gender, age, and grade level influence reflective learning. Sargent (2015) found that males reflect more in business courses, while females reflect more in humanities and science courses. Aqadoh and Trimasse (2024) found that the higher levels of education can enhance reflective thinking. Furthermore, LPA can also support dynamic prediction (Yalçin et al., 2021). Based on this, we propose the Hypothesis 2: gender, age, and grade level can influence latent types of reflective learning.

### 2.3 Feedback literacy and reflective learning

In the feedback model proposed by Hattie and Clarke (2018), feedback is regarded as a dynamic cognitive activation process that can trigger learners' reflection in action, thereby facilitating deep learning. Specifically, firstly, strong feedback literacy can use feedback to break students' ingrained habitual thinking. Carless and Boud (2018) pointed out that feedback can help students recognize the limitations in their knowledge acquisition and skill application, thereby breaking established thinking patterns and prompting them to adjust their learning strategies. Yilmaz's (2020) empirical study also demonstrates that methods combining real-time analysis with personalized feedback enable students to better monitor their learning trajectories, continuously adjust learning behaviors through feedback information, and enhance learning outcomes. Secondly, strong feedback literacy fosters reflective comprehension, thereby enabling individuals to more effectively engage with critiques and embrace suggestions. Carless and Boud (2018) noted that feedback enhances students' empathy by clarifying their strengths and weaknesses, enabling them to better understand the intentions and expectations of feedback providers. Pieper et al. (2021) found that providing high-information feedback in reflective journals, including in-depth reflections on concepts and perspectives, significantly enhances students' engagement with the learning process and depth of understanding. Thirdly, strong feedback literacy enables deep reflection grounded in understanding, allowing individuals to recognize the significance and value of critiques and suggestions. Zhang et al. (2024) argue that feedback literacy, as a form of evaluative discourse, functions by exposing discrepancies between students' self-perceptions and external expectations, thereby inducing cognitive dissonance. To alleviate this discomfort, students will actively reflect on critiques and suggestions in feedback, aligning them with their own contexts, thereby recognizing their significance and value. Empirical studies also substantiate this perspective. Mauri and Neiva de Figueiredo (2025) discovered that a dual-loop peer feedback system amplifies reflective outcomes through iterative dialogue and collaborative revision; Wang et al. (2024), in their study integrating virtual reality (VR), feedback mechanisms, and ChatGPT, found that this combined approach enhanced students' reflective thinking through real-time feedback. Finally, strong feedback literacy enables critical reflection built upon introspection, leading to the transformation of pre-existing beliefs. Brookfield (1996) argued that feedback offers an external perspective to help students identify blind spots and engage in cognitive reappraisal, thereby fostering critical thinking and strengthening self-awareness. Chen et al. (2024) found in their empirical study that feedback promotes the development of critical thinking by triggering cognitive conflicts and activating metacognition. In summary, feedback progressively guides learners toward deeper thinking by breaking established thinking patterns, fostering reflective comprehension, and enabling critical reflection, thereby prompting them to continuously adjust and optimize their learning strategies. This process ultimately leads to significant improvements in learning outcomes and sustained self-development. Based on this, the study proposes Hypothesis 3: feedback literacy has a significant impact on the reflective learning types of Chinese high school students.

### 2.4 Reflective learning and academic performance

Reflective learning, defined as the deliberate process of analyzing experience to reconstruct knowledge and guide future actions (Schön, 1983), can promote deep cognitive engagement and consequently enhance academic performance. For example, Loka et al. (2019) demonstrated that both reflection and critical reflection significantly improve academic outcomes by enabling students to systematically evaluate and adjust learning strategies. Different forms of reflection, such as specific reflective prompts and good reflective writing skills, can further improve academic performance (Menekse et al., 2022; Tsingos-Lucas et al., 2017). Additionally, reflective motivation amplifies academic success by increasing engagement and persistence in complex tasks (Cavilla, 2017; Wang et al., 2023). In Chinese high school education system, which adopts a teaching model of liberal arts and science separation, subjects like Chinese, Mathematics, and English are common and representative in assessing academic performance. Therefore, Hypothesis 4 is proposed: different latent types of reflective learning affect academic performance in different subjects.

## 3 Methods

### 3.1 Research design

This study employed a cross-sectional research design, utilizing the statistical technique of Latent Profile Analysis (LPA) to accomplish the research objectives. First, data were collected through the Reflective Thinking Questionnaire, Feedback Literacy Scale, and a demographic survey, and standardized test scores. Second, LPA was applied to identify distinct subgroups based on the reported levels of reflective learning. Finally, the subsequent analysis of LPA were conducted. Gender, age, grade, and feedback literacy were included as predictors to examine their associations with reflective learning profiles using the *R3STEP* method; The *BCH* method was employed to quantify the impact of reflective learning profiles on academic performance across Chinese, Mathematics, and English.

### 3.2 Participants

A total of 720 students from an urban public high school in Shanxi Province, China, were initially recruited through stratified random sampling by grade (Senior 1 to Senior 3). The school draws junior high school graduates from various urban districts, counties, and townships, ensuring a representative sample. This study adheres to the principle of voluntary student participation. Sixteen participants were excluded due to invalid questionnaires (e.g., patterned responses), missing values, and outliers, resulting in a final sample of 704 students (*M* = 17.38, *SD* = 1.089), yielding a 97.78% valid response rate. The sample included 385 female students (*M* = 17.32, *SD* = 1.075) and 319 male students (*M* = 17.45, *SD* = 1.103), distributed across grades as follows: 306 in Senior 1 (*M* = 16.47, *SD* = 0.606), 202 in Senior 2 (*M* = 17.6, *SD* = 0.657), 196 in Senior 3 (*M* = 18.58, *SD* = 0.671).

### 3.3 Research instruments

#### 3.3.1 Reflective thinking questionnaire (LRTQ)

This study utilized the LRTQ developed by Kember et al. (2000) to measure the reflective learning capacities of high school students. The questionnaire includes four dimensions: habitual behavior, understanding, reflection and critical reflection, comprising a total of 16 items. Using a 5-point Likert scale (1 = “completely agree”, 5 = “completely disagree”). Higher scores in each dimension suggest a higher level of reflective learning. *Cronbach's Alpha* was 0.893 and *KMO* was 0.918.

#### 3.3.2 Feedback literacy scale (FLS)

This study employed the Chinese version of the FLS developed by Zhan (2022). The scale includes six dimensions: elicitation, processing, enacting, appreciation, readiness and commitment, comprising a total of 24 items. Using a 6-point Likert scale (1 = “strongly disagree”, and 6 = “strongly agree”). Higher scores in each dimension indicate a better level of feedback literacy among students. *Cronbach's Alpha* was 0.967 and *KMO* was 0.959.

#### 3.3.3 Chinese, Mathematics, and English test papers

Unified test papers with good discrimination and difficulty levels were used. Each subject had a full mark of 150 points. Objective questions were scored by a scanning machine, and subjective questions were scored by two subject teachers, with the average score taken as the final result. A higher score indicates better academic performance.

### 3.4 Procedure

The specific research procedures of this study are as follows: first, preparation of materials and ethical review. The research team prepared the materials and submitted them to the Ethics Committee of the Key Laboratory of Modern Teaching Technology, Ministry of Education, Shaanxi Normal University. Formal approval was obtained (Approval No.: L20230210-01). The materials included instructions, demographic questionnaires, Feedback Literacy Scale, Reflective Thinking Questionnaire, and consent forms for students and parents. Second, determine the participants using stratified cluster random sampling, choosing seven classes of first-year, four classes of second-year, and four classes of third-year high school students. After obtaining consent from students and parents and signing informed consent forms, 720 participants were formally enrolled. Third, survey implementation and data collection. Under the supervision of a doctoral candidate in Curriculum and Instruction from Shaanxi Normal University, with assistance from homeroom teachers, participants completed the *QuestionStar* online questionnaires. Standardized test scores for Chinese, Mathematics, and English were collected 2 weeks post-survey. Finally, researchers organized and analyzed the data to obtain results.

### 3.5 Statistical analyses

This study employed *SPSS* 22.0 and *Mplus* 7.4 for data management and analysis. The main steps included: (1) *SPSS* 22.0 was used to conduct common method bias tests, normality tests, descriptive statistics, and correlation analyses to examine the validity of the data; (2) Latent Profile Analysis (LPA) was implemented in *Mplus* 7.4 to identify distinct latent types of reflective learning, thereby testing Hypothesis H1. The study used Akaike's Information Criterion (*AIC*), Bayesian Information Criterion (*BIC*), Sample-size-adjusted Bayesian Information Criterion (*SSA-BIC*), Entropy, Lo-Mendell-Rubin Likelihood Ratio Test (*LMR*), and Bootstrap Likelihood Ratio Test (*BLRT*) as reference indicators to compare the fit of different latent class models. Lower *AIC, BIC*, and *SSA*-*BIC* values indicate better model fit; higher Entropy indicates better classification accuracy (values above 0.8 mean over 90% accuracy); *LMR* and *BLRT p*-values below 0.05 show the n-class model is better than the n-1-class model; and (3) Subsequent analyses based on the LPA results were conducted. First, taking the reflective learning latent types as the dependent variable, the *R3STEP* method was used to test their relationships with gender, age, grade, and feedback literacy, Odds ratios (OR) were analyzed for significance and magnitude to evaluate Hypotheses H2 and H3. Odds Ratios (*OR*) indicate the likelihood of students belonging to one latent pattern vs. another based on predictive variables. Second, taking the reflective learning latent types as the independent variable, the *BCH* method was used to test differences in academic performance in Chinese, Mathematics, and English, The χ^2^ test, *post-hoc* pairwise comparisons, and their significance levels were used to test Hypothesis H4.

## 4 Research results

### 4.1 Test of common method bias

To clarify whether the survey data were influenced by social desirability effects, this study employed Harman's single-factor analysis to conduct a common method bias test. The unrotated factor analysis of all items using *SPSS* 22.0 showed that there were seven factors with eigenvalues exceeding 1, among which the first factor accounted for 38.068% of the total variance, below the 40% threshold. This indicated that there is no significant common method bias in this study, and the collected data are valid for subsequent analysis.

### 4.2 Correlation analysis of key variables

This study used *SPSS* 22.0 to conduct normality tests, descriptive statistics, and correlation analyses for all variables and their internal factors (Table 1). The one-sample Kolmogorov-Smirnov test results indicated that all variables conformed to a normal distribution. Key findings from the correlation analysis revealed that gender (1 = male, 2 = female) was not correlated with age, grade, feedback appreciation, feedback commitment, understanding, and Mathematics scores, but was positively correlated with Chinese and English scores, and negatively correlated with the remaining variables. Age was not correlated with feedback commitment or reflection, but was positively correlated with grade and negatively correlated with the remaining variables. Grade was negatively correlated with the remaining variables. Feedback literacy and its internal factors were positively correlated with reflective learning and its internal factors.

**Table 1:** Descriptive statistics and correlation analysis of key variables (*n* = 704).

**Variables**	***M***	***SD***	**1**	**2**	**3**	**4**	**5**	**6**	**7**	**8**	**9**	**10**	**11**	**12**	**13**	**14**	**15**	**16**
Gender	1.55	0.498	1															
Age	1.84	0.830	−0.034	1														
Grade	17.38	1.089	−0.060	0.810^***^	1													
Feedback elicitation	4.40	0.829	−0.124^***^	−0.114^**^	−0.145^***^	1												
Feedback processing	4.39	0.801	−0.075^*^	−0.080^*^	−0.101^***^	0.708^***^	1											
Feedback enacting	4.26	0.845	−0.092^*^	−0.100^**^	−0.132^***^	0.708^***^	0.746^***^	1										
Feedback appreciation	4.45	0.795	−0.042	−0.077^*^	−0.098^**^	0.667^***^	0.747^***^	0.749^***^	1									
Feedback readiness	4.26	0.901	−0.084^*^	−0.124^***^	−0.116^**^	0.574^***^	0.623^***^	0.573^***^	0.667^***^	1								
Feedback commitment	4.43	0.761	−0.044	−0.035	−0.085^*^	0.639^***^	0.657^***^	0.698^***^	0.719^***^	0.674^***^	1							
Habitual action	2.76	0.728	−0.114^***^	−0.221^***^	−0.183^***^	0.198^***^	0.164^***^	0.223^***^	0.132^***^	0.207^***^	0.160^***^	1						
Understanding	3.40	0.618	−0.067	−0.076^*^	−0.112^**^	0.439^***^	0.428^***^	0.414^***^	0.413^***^	0.423^***^	0.475^***^	0.346^***^	1					
Reflection	3.41	0.626	−0.137^***^	−0.069	−0.082^*^	0.439^***^	0.402^***^	0.428^***^	0.410^***^	0.407^***^	0.451^***^	0.373^***^	0.731^***^	1				
Critical reflection	3.22	0.643	−0.122^***^	−0.181^***^	−0.165^***^	0.435^***^	0.369^***^	0.424^***^	0.389^***^	0.415^***^	0.444^***^	0.484^***^	0.673^***^	0.703^***^	1			
Chinese scores	93.11	9.939	0.075^*^	−0.285^***^	−0.294^***^	0.056	0.064	0.062	0.070	0.095^*^	0.083^*^	0.076^*^	0.085^*^	0.055	0.090^*^	1		
Mathematics scores	73.85	23.821	−0.069	−0.296^***^	−0.289^***^	0.120^***^	0.077^*^	0.127^***^	0.083^*^	0.067	0.087^*^	0.172^***^	0.057	0.108^**^	0.114^**^	0.432^***^	1	
English scores	91.31	23.830	0.213^***^	−0.286^***^	−0.309^***^	0.122^***^	0.117^**^	0.087^*^	0.135^***^	0.099^**^	0.151^***^	0.025	0.142^***^	0.100^**^	0.112^**^	0.481^***^	0.417^***^	1

* *p* < 0.05.

** *p* < 0.01.

*** *p* < 0.001.

### 4.3 Latent profile analysis of reflective learning

This study employed *Mplus* 7.4 to perform a Latent Profile Analysis on high school students' reflective learning. To determine the optimal grouping model, the analysis began with a two-group model and progressively added one group at a time until the best grouping configuration was identified (Table 2).

**Table 2:** Latent Profile Analysis results of reflective learning among high school students (*n* = 704).

**Model**	**k**	**AIC**	**BIC**	**SSA-BIC**	**Entropy**	**LMR (*p*)**	**BLRT (*p*)**	**Class probability**
2C	25	25,773.504	25,996.786	25,841.200	0.918	<0.0001	<0.0001	0.688/0.312
3C	34	25,142.517	25,443.265	25,233.701	0.929	0.0001	<0.0001	0.609/0.338/0.053
**4C**	**83**	**24,929.861**	**25,308.073**	**25,044.530**	**0.839**	**0.0427**	**<0.0001**	**0.229/0.156/0.463/0.152**
5C	100	24,731.432	25,187.109	24,869.588	0.837	0.3356	<0.0001	0.011/0.220/0.418/0.300/0.051

Bold values indicate the optimal latent profile type.

**Figure 1:**
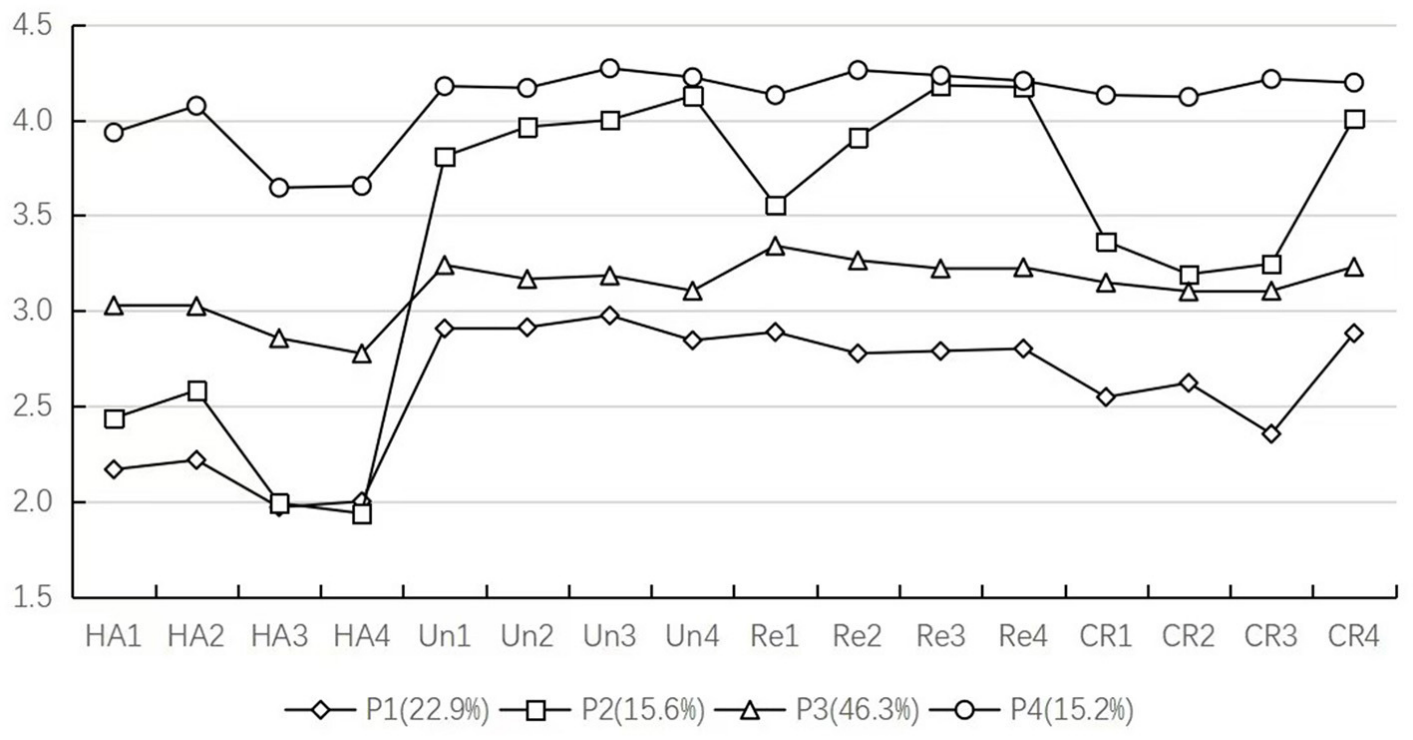
The four-class latent profiles model of reflective learning among high school students. HA, habit action; UN, understanding; RE, reflection; CR, critical reflection.

Table 2 presents the model fitting indices for Latent Profile Analysis. As the number of classes increased, *AIC, BIC*, and *SSA-BIC* values progressively decreased, indicating that the more classes there are, the better the model fits. Consequently, the 2C model was excluded. The 5C model was also rejected due to a non-significant *LMR p*-value (*p* > 0.05). Both the 3C and 4C models demonstrated Entropy values above 0.8 and significant *LMR* and *BLRT p*-values (*p* < 0.05). However, in the 3C model, the proportions of each latent profile group were 60.9%, 33.8%, and 5.3% respectively, with the third latent profile group accounting for less than 10% (close to 5%; Figure 1), indicating instability. In contrast, the 4C model exhibited balanced proportions (all >10%) and superior fit across indices, thus appropriately representing the characteristics of reflective learning types of high school students. Based on the fit criteria and substantive relevance, the 4C model was selected as the optimal latent profile analysis model. The findings supported Hypothesis H1.

In this study, the level of reflective thinking questionnaire employed a 5-point Likert format with a median score of 3. Each profile in the 4C model was named based on the mean scores of habit action, understanding, reflection, and critical reflection behaviors (Figure 1). Group P1 had 161 participants (22.9%) with the mean values of each dimension being 2.09, 2.91, 2.81, and 2.60. This group had low habit action and critical reflection, with moderate understanding and reflection (below the median). It was named the Low Reflective Learning Group (LRLG). Group P2 had 110 participants (15.6%) with the mean values of each dimension being 2.24, 3.98, 3.95, and 3.45. This group had low habit action, high understanding and reflection, intermediate critical reflection, and showed notable instability in reflection and critical reflection. It was named the Low Habits-High Understanding Learning group (LHHULG). Group P3 had 326 participants (46.3%) with the mean values of each dimension being 2.92, 3.17, 3.26, and 3.15. This group had moderate levels in all aspects and was named the Moderate Reflective Learning Group (MRLG). Group P4 had 107 participants (15.2%) with the mean values of each dimension being 3.83, 4.21, 4.21, and 4.17. This group was at a high level in all aspects and was named the High Reflective Learning Group (HRLG; Table 3).

**Table 3:** The names and abbreviations of high school students' reflective learning latent profile types.

**Group**	**Group names**	**Abbreviation**
P1	Low reflective learning group	LRLG
P2	Low habits-high understanding learning group	LHHULG
P3	Moderate reflective learning group	MRLG
P4	High reflective learning group	HRLG

### 4.4 The relationship between demographic variables, feedback literacy, and latent types of reflective learning

This study utilized *Mplus* 7.4 for the subsequent analysis of LPA, investigating the relationship between predictive variables (such as age, gender, grade, and feedback literacy) and the latent profile types of reflective learning among high school students (see Table 4). Odds Ratios (*OR*) indicate the likelihood of students belonging to one latent pattern vs. another based on predictive variables. For instance, an *OR* value of 2.430 suggests that individuals who receive better feedback are 2.43 times more likely to belong to the P4 (HRLG) than to the P1 (LRLG).

**Table 4:** Logistic regression results of age, gender, grade, and feedback literacy dimensions on the four reflective learning types.

**Variables**	**P2 vs. P1**		**P3 vs. P1**		**P4 vs. P1**		**P3 vs. P2**		**P4 vs. P2**		**P4 vs. P3**	
	**Coef**.	**OR**	**Coef**.	**OR**	**Coef**.	**OR**	**Coef**.	**OR**	**Coef**.	**OR**	**Coef**.	**OR**
Gender	**−0.895**	**0.409**	**−0.615**	**0.541**	**−1.014**	**0.363**	0.28	1.323	−0.119	0.888	−0.399	0.671
Age	−0.275	0.760	−0.259	0.772	**−0.465**	**0.628**	0.016	1.016	−0.191	0.826	−0.207	0.813
Grade	0.038	1.039	−0.212	0.809	−0.512	0.599	−0.25	0.779	**−0.552**	**0.576**	−0.302	0.739
Feedback elicitation	0.408	1.504	**0.272**	**1.313**	**0.888**	**2.430**	−0.136	0.873	0.481	1.618	**0.616**	**1.852**
Feedback processing	0.198	1.219	−0.11	0.896	−0.178	0.837	−0.308	0.735	−0.376	0.687	−0.068	0.934
Feedback enacting	0.074	1.077	**0.534**	**1.706**	**0.849**	**2.337**	0.46	1.584	0.775	2.171	0.316	1.372
Feedback appreciation	0.107	1.113	−0.409	0.664	−0.592	0.553	−0.516	0.597	−0.699	0.497	−0.183	0.833
Feedback readiness	**0.713**	**2.040**	**0.64**	**1.896**	**0.799**	**2.223**	−0.073	0.930	0.086	1.090	0.159	1.172
Feedback commitment	0.400	1.492	−0.071	0.931	0.642	1.900	−0.471	0.624	0.242	1.274	**0.713**	**2.040**

Coef., coefficient; OR, odds ratio; male students and grade 1 were used as the baseline group. Bold denotes the significance of Coef. and OR values.

The results showed that gender, age, and grade level significantly influence the latent types of reflective learning (Table 4). The younger and the lower grades are more likely to be classified into the P4 (HRLG). Males tend to excel in P2 (LHHULG), P3 (MRLG), and P4 (HRLG), while females are more adept at P1 (LRLG). Specifically, as age increases, the proportion of students in P4 (HRLG) significantly decreases compared to P1 (LRLG). With the increase of grade level, the proportion of students in P4 (HRLG) significantly decreases compared to P2 (LHHULG). Compared to males, females are less likely to be in P2 (LHHULG), P3 (MRLG), and P4 (HRLG) than in P1 (LRLG). The results of this study supported Hypothesis H2.

Additionally, the results showed that feedback elicitation, enacting, readiness, and commitment affected reflective learning types, while feedback processing and appreciation did not. As the level of feedback elicitation increased, the proportion of students in the P3 (MRLG) and P4 (HRLG) significantly increased, with the number of students in the P4 (HRLG) exceeding than that in the P3 (MRLG). As the level of enacting increased, the proportion of students in the P3 (MRLG) and P4 (HRLG) significantly increased. As the level of feedback readiness increased, the proportion of students in the P2 (LHHULG), P3 (MRLG), and P4 (HRLG) significantly increased. As the level of feedback commitment increased, the number of students in the P4 (HRLG) was significantly greater than that in the P3 (MRLG). Some of the results from this study supported Hypothesis H3.

### 4.5 Comparative analysis of the differences in academic performance among high school students based on four latent types of reflective learning

This study utilized *Mplus* 7.4 for the subsequent analysis of LPA, investigating the relationship between outcome variables (such as Mathematics scores, English scores, and Chinese scores) and the latent profile types of reflective learning among high school students (see Table 5). The results revealed that the four latent types of reflective learning affected Mathematics scores (χ^2^ = _21.613_, *p* < 0.001) and English scores (χ^2^= 12.135, *p* < 0.01), but not in Chinese scores (χ^2^= 6.320, *p* > 0.05; see Table 5). *Post-hoc* pairwise comparisons revealed that among the effects of latent types of reflective learning on high school students' Mathematics scores, the P4 (HRLG) performs the best, followed by the P3 (MRLG) and the P2 (LHHULG) with no significant difference between them, while the P1 (LRLG) performs the worst. For English scores, P1 (LRLG) is significantly lower than P2 (LHHULG) and P4 (HRLG), with no significant difference observed between P1 (LRLG) and P3 (MRLG). The P3 (MRLG) is significantly lower than P4 (HRLG), while no significant differences are found between P3 (MRLG) and P2 (LHHULG), or P2 (LHHULG) and P4 (HRLG). Some of the results from this study supported Hypothesis H4.

**Table 5:** Comparison of differences in Chinese, Mathematics, and English scores among four latent types of reflective learning (*n* = 704).

**Variables**	**P1**		**P2**		**P3**		**P4**		**BCH (X2)**	**P1 vs. P2**	**P1 vs. P3**	**P1 vs. P4**	**P2 vs. P3**	**P2 vs. P4**	**P3 vs. P4**
	***M***	***SE***	***M***	***SE***	***M***	***SE***	***M***	***SE***							
Chinese scores	91.50	0.910	93.74	0.92	93.16	0.610	94.87	1.096	6.320	2.999	1.953	5.594	0.260	0.589	1.828
Mathematics scores	67.01	1.943	74.70	2.166	74.59	1.526	81.58	2.575	21.613^***^	6.997^**^	7.998^***^	7.998^**^	0.002	3.993^*^	5.378^*^
English scores	88.52	2.096	94.69	2.089	89.69	1.567	97.03	2.294	12.135^**^	4.348^**^	0.172	7.520^**^	3.386	0.543	6.865^**^

* *p* < 0.05.

** *p* < 0.01.

*** *p* < 0.001.

## 5 Discussion

### 5.1 Latent profile analysis of reflective learning among high school students

The research findings supported Hypothesis H1. Through Latent Profile Analysis (LPA), high school students are categorized into four latent types of reflective learning: the Low Reflective Learning Group (LRLG), the Low Habit-High Understanding Learning Group (LHHULG), the Moderate Reflective Learning Group (MRLG), and the High Reflective Learning Group (HRLG). The MRLG has the largest number of individuals, followed by the LRLG and LHHULG, with the HRLG having the fewest. This classification differs from the findings of Radović et al. (2023), as this research employs LPA to categorize and describe the characteristics of the types.

Each type shows unique features and significant differences in the four dimensions of habitual behavior, understanding, reflection, and critical reflection. Specifically, students in the LRLG lack basic study habits and the ability to integrate knowledge. They don't think deeply or critically, tending to think and learn superficially. Students in the LHHULG lacks thinking habits and critical scrutiny but demonstrates strength in knowledge integration, problem identification, and resolution. These students have obvious thinking preferences. Students in the MRLG demonstrates moderate performance across thinking habits, knowledge construction, problem-solving, and critical innovation, exhibiting limited depth in these cognitive domains. Students in the HRLG have better thinking habits. They excel at building a web-like knowledge system and at discovering, representing, and solving problems. They are also skilled in critical thinking, constantly improving concepts and innovating. They show excellent qualities like thinking ahead, investigating and monitoring during tasks, and improving afterwards. Students in the HRLG exhibits sound thinking habits, excelling in constructing knowledge frameworks, identifying and solving problems, and engaging in critical thinking to continuously refine concepts and achieve innovation, demonstrating strengths in pre-task deliberation, in-process inquiry and monitoring, and post-task refinement. The four latent profile types reflect the characteristics of reflective learning subgroups. This helps teachers provide targeted reflective learning guidance, enabling students to learn effectively and improve learning efficiency.

### 5.2 The relationship between ages, grades, genders and the latent types of reflective learning among high school students

The results supported Hypothesis 2: age, grade, and gender can influence changes in reflective learning latent types. Some of the results of this study are consistent with previous research, while others diverge. First, this study found that younger students are more likely to be categorized into the HRLG, which is inconsistent with Sargent's (2015) findings. As students grow older, they accumulate more experience of transforming uncertain situations into determinate ones, and their reflective cognitive demand consequently diminishes (Sladek et al., 2010). At this stage, substantial cognitive resources are no longer required for deep thinking. Second, with advancing grade level, there are fewer students in the HRLG, while more in the LHHULG, which is inconsistent with the research of Aqadoh and Trimasse (2024). There are two reasons for the above. On the one hand, as grade level increases, students acquire more scientific knowledge, and many uncertain situations are resolved due to the acquisition of knowledge, thus the number of deep reflection individuals decreases; On the other hand, influenced by the division of arts and sciences in high school, senior students not only develop preferences in learning content but also in thinking forms, placing more emphasis on the practical value of understanding and reflective behaviors. Last, males tend to HRLG, MRLG, and LHHULG, while females tend to LRLG. The influence role of gender in reflective learning is confirmed once again (Sargent, 2015). Frederick (2005) used the cognitive reflection test (CRT) to reveal gender differences, showing that males perform more prominently in cognitive reflection abilities, tending toward reflective thinking, while females are more inclined toward intuitive and experience-driven cognitive styles.

### 5.3 The relationship between feedback literacy and the latent types of reflective learning

The research findings partially supported Hypothesis 3: among feedback literacy components, feedback elicitation, enacting, commitment, and readiness significantly influenced reflective learning types among high school students, while feedback processing and appreciation did not. This finding can be explained by the feedback model proposed by Hattie and Clarke (2018) and the theoretical framework of Zhan (2022). Feedback elicitation focuses on seeking diverse external support, enabling students to acquire criteria for evaluating the quality of learning actions, identify gaps between goals and outcomes or between design and implementation, and thereby clarify and resolve problems. Feedback enacting fundamentally requires creating an actionable framework that activates students' improvement-oriented rational actions. This involves controlled, goal-directed inquiry processes guided by explicit objectives and concrete designs, following the cognitive pathway from contextual analysis through problem identification, hypothesis formulation, logical deduction, to empirical verification. Feedback readiness embodies a proactive approach to confronting challenges, ensuring students dare to acknowledge mistakes, accept external perspectives, courageously undertake cross-disciplinary challenges, and persistently and effectively implement reflective practices. Feedback commitment embodies decisive, rational, adaptive, and persistent volition, sustaining students' reflective momentum and motivating continuous transformation through iterative cycles of practice, reflection, re-practice, and re-reflection. Collectively, the theoretical roles of feedback elicitation, enacting, readiness, and commitment align with empirical findings in shaping reflective learning profiles. However, this study found that feedback processing and appreciation had no significant effects on reflective learning types. Theoretically, Feedback processing enables students to critically evaluate evaluative discourse, extract key insights from identified gaps, and implement targeted improvements. In reality, most individuals struggle to accept evaluative discourse, particularly negative feedback. Moreover, high school students in adolescence often exhibit rebellious tendencies, potentially intensifying their resistance to critical commentary. Feedback appreciation, which emphasizes perspective-taking and assimilating suggestions, helps students re-examine issues from alternative viewpoints and adopt optimal strategies. In fact, due to the cognitive inertia of “being too involved to see clearly” (a phenomenon where one's close engagement blinds them to external perspectives), most individuals find it challenging to internalize others' critiques. The above explanation may be the main reason why feedback processing, feedback appreciation, and reflective learning types are unrelated. It also cautions feedback providers to be mindful of their approach when offering “critical comments” and “effective suggestions” to those receiving feedback.

### 5.4 The relationship between the latent types of reflective learning and academic performance

The research results partially supported Hypothesis 4: the latent types of reflective learning have a significant impact on Mathematics and English scores, but have little effect on Chinese scores. In terms of Mathematics, students in the HRLG perform the best. This finding is consistent with the research of Büscher and Prediger's (2019), who pointed out that reflective learning can enhance students' mathematical literacy, especially in solving practical problems. In other words, reflective learning helps improve Mathematics scores by creating reflective learning situations that help students connect mathematical knowledge with real-world problems, thereby enhancing their mathematical literacy and problem-solving abilities. Regarding English performance, for Chinese students, English language learning involves a second language acquisition process. The study by Anani Sarab and Mardian (2023) suggests that reflective learning plays a significant role in enhancing second language acquisition by fostering deeper engagement with language rules and promoting autonomous learning strategies. This corresponds with the findings of the current study, where reflective learning was shown to positively impact on English language performance by prompting students to think about their learning, thereby improving their understanding of language structures and promoting more independent learning behaviors. However, in terms of Chinese scores, the latent types of reflective learning did not show significant differences. Perhaps we can explain it in this way: different learning tasks can affect strategy selection (Onan et al., 2024), and students may not have employed reflective learning strategies in the Chinese language subject. The Chinese language discipline focuses on the cultivation of humanistic literacy, which requires long-term accumulation and internalization, Cavilla's (2017) also suggested that short-term reflection activities may primarily affect students on an emotional rather than cognitive level. This might be one of the reasons why there is no direct connection between Chinese grades and reflective learning.

### 5.5 Limitations and prospects

The limitations of this study are as follows: first, the subject sample range is relatively narrow and the coverage is small, which may limit the theoretical applicability of the research findings. Future studies could expand the research scope and increase the sample size to conduct cross-regional and cross-cultural studies. Second, this study only uses cross-sectional research data and failed to fully explore the development changes of latent types. Future studies could employ longitudinal tracking data to analyze transformation of latent profiles. Third, this study does not empirically verify why feedback processing and appreciation do not lead to the transformation of reflective learning types. Future studies could deeply explore the impact of feedback processing and feedback appreciation in reflective learning.

## 6 Conclusion

This study, through Latent Profile Analysis, discovered that high school students' reflective learning exists in four latent profiles: the high reflective learning group (HRLG), the moderate reflective learning group (MRLG), the low habit-high understanding learning group (LHHULG), and the low reflective learning group (LRLG), each of which has its own uniqueness. As age and grade level increase, the number of students in the HRLG decreases; males tend to HRLG, MRLG and LHHULG, while females tend toward LRLG. The research reveals that feedback literacy plays a promotional role in types of reflective learning and emphasize the key functions of feedback elicitation, enacting, commitment, and readiness within feedback literacy. The four types of reflective learning can influence Mathematics and English achievement. These findings refine the conceptualization of reflective learning types and enhance the understanding of how feedback literacy affects these types. From a practical standpoint, the research results offer insights for educational reforms aimed at improving students' comprehensive qualities, especially in integrating feedback literacy into teaching to promote deeper reflection and more effective learning strategies.

## 7 Inspiration

Based on the aforementioned research, we can draw the following insights: first, the existence of the four types of reflective learning is objective, and teachers should conduct differentiated teaching for students of different types. For students in the Low Reflective Learning Group, teachers can provide clear thinking frameworks and guidance, and set specific thinking tasks and questions. For students in the Low Habit-high Understanding Learning Group, teachers can design diverse learning activities to stimulate their interest in thinking and encourage them to explore their own thinking preferences. For students in the Moderate Reflective Learning Group, group discussions, role-playing, and other methods can be used to cultivate students' autonomy and creativity. For students in the High Reflective Learning Group, teachers should encourage them to engage in higher-level critical thinking to promote the development of their innovation and lifelong learning abilities. Second, under natural conditions, students' high reflective learning abilities tend to decrease with increasing age and grade level. Teachers should take proactive measures to counteract this trend and enhance students' deep reflective learning capabilities. Third, teachers should focus on guiding and cultivating students' abilities to actively seek feedback, make effective plans, prepare for challenges, and maintain a strong determination to reflect, thereby enhancing the role of feedback literacy in transforming reflective learning types. Fourth, students with high reflective learning abilities tend to have better grades. Teachers should consider transforming reflective learning types as a means to improve academic performance.

## Data Availability

The original contributions presented in the study are included in the article/supplementary material, further inquiries can be directed to the corresponding author.
